# Microbiome Shifts Associated With the Introduction of Wild Atlantic Horseshoe Crabs (*Limulus polyphemus*) Into a Touch-Tank Exhibit

**DOI:** 10.3389/fmicb.2020.01398

**Published:** 2020-07-14

**Authors:** Ariel D. Friel, Sean A. Neiswenter, Cale O. Seymour, Lauren Rose Bali, Ginger McNamara, Fabian Leija, Jack Jewell, Brian P. Hedlund

**Affiliations:** ^1^School of Life Sciences, University of Nevada, Las Vegas, NV, United States; ^2^Shark Reef Aquarium at Mandalay Bay, Las Vegas, NV, United States; ^3^Nevada Institute of Personalized Medicine, University of Nevada, Las Vegas, NV, United States

**Keywords:** 16S rRNA, aquarium, captivity, bacteria, marine, holobiont

## Abstract

The Atlantic horseshoe crab (*Limulus polyphemus*) is a common marine aquarium species and model organism for research. There is potential monetary and conservation value in developing a stable captive population of horseshoe crabs, however, one major impediment to achieving captivity is a lack of knowledge regarding captive diseases. We utilized 16S rRNA gene amplicon sequencing to track changes in the microbiomes of four body locations in three wild-caught (tracked over 14 months in captivity) and three tank-acclimated (>2 years in captivity) adult *L. polyphemus* in a touch tank at Shark Reef Aquarium at Mandalay Bay in Las Vegas, NV. The wild population hosted diverse and distinct microbiomes on the carapace (260 ± 96 amplicon sequence variants or ASVs), cloaca (345 ± 77 ASVs), gills (309 ± 36 ASVs), and oral cavity (359 ± 37 ASVs), which were dominated by classes *Gammaproteobacteria*, *Bacteroidia*, and *Alphaproteobacteria*. A rapid decline in richness across all body locations was observed within 1 month of captivity, with tank-acclimated (>2 years) animals having <5% of the initial microbiome richness and a nearly completely restructured microbial community. Tank-acclimated horseshoe crabs possessed distinct microbiomes that were highly uneven and low in species richness on the carapace (31 ± 7 ASVs), cloaca (53 ± 19 ASVs), gills (17 ± 2 ASVs), and oral cavity (31 ± 13 ASVs). The carapace, oral cavity, and gills of the tank-acclimated animals hosted abundant populations of *Aeromonas* (>60%) and *Pseudomonas* (>20%), both of which are known opportunistic pathogens of aquatic animals and can express chitinases, providing a plausible mechanism for the development of the carapace lesion pathology observed in this and other studies. The cloaca of the tank-acclimated animals was slightly more diverse than the other body locations with *Aeromonas*, *Enterococcus*, *Shewanella*, and *Vagococcus* dominating the community. These results provide an important baseline on the microbiomes of both wild and tank-acclimated horseshoe crabs and underscore the need to continue to investigate how native microbial populations may protect animals from pathogens.

## Introduction

Perhaps the most challenging environmental change an organism can experience is when a wild individual is taken from a natural setting and held in captivity under artificial conditions as is the case in a laboratory, the pet trade, the food industry, or zoos and aquaria. Replicating the natural environment is nearly impossible under artificial conditions and depending on the circumstances it may be necessary or more convenient to modify conditions, such as temperature or salinity, from those an organism would experience in the wild. Additionally, although some conditions might be controlled to limit variability under captive conditions, other variables might cycle or build up to unnatural levels in captivity, such as the case with dissolved nitrogen in aquaculture (Hargreaves, 1998). In captivity, an organism may also be exposed to population densities and different species they would never encounter in natural settings, fostering novel biotic interactions (Morgan and Tromborg, 2007). This can be particularly pronounced for aquatic organisms, such as in aquaculture or large aquaria, where high densities of a variety of host species share the same tank or have a common source of filtered water. In such artificial systems stress can lead to microbiome dysbiosis and infections by obligate or opportunistic pathogens (Llewellyn et al., 2014).

*Limulus polyphemus*, the Atlantic horseshoe crab, is one of four species in the order *Xiphosurida* and the only species in North America. Horseshoe crabs have a deep evolutionary history dating over 400 mya to at least the Ordovician (Rudkin and Young, 2009). Contrary to what their common name implies, horseshoe crabs are not crustaceans, but represent a highly divergent lineage that is more closely related to sea spiders and other arachnids than crabs (Ballesteros and Sharma, 2019). *L. polyphemus* is widespread along the continental shelf of North America’s east coast and occupies a broad range of temperature and salinity (Sekiguchi and Shuster, 2009). Adults are strictly benthic and burrow through sediments to feed on polychaetes, bivalves, and other benthic fauna (Botton, 1984, 2009). Given their deep evolutionarily history (Kin and Blażjowski, 2014) and highly variable ecology it is likely that horseshoe crabs harbor a unique and diverse microbiome.

*Limulus polyphemus* have considerable value both commercially and ecologically. The pharmaceutical industry utilizes their blood to produce *Limulus* amebocyte lysate, which is used to detect endotoxins and for bait by the commercial eel industry. *L. polyphemus* can also serve as a model organism for a variety of research topics, including embryology and vision research (Liu and Passaglia, 2009; Williams, 2019). Additionally, millions of migratory birds refuel on their eggs each spring at spawning sites (Niles et al., 2009). As a highly unusual and non-threatening organism, they are also used to garner interest and educate the general public through interactive exhibits at aquaria.

Due to multifaceted threats, horseshoe crab populations have been in decline (Smith et al., 2017). There is a potential utility to multiple stakeholders in the maintenance of captive populations (Carmichael and Brush, 2012). However, very little is known of the microbial communities of either healthy or diseased horseshoe crabs. A common affliction of wild and captive horseshoe crabs is shell disease, which presents itself as carapace discoloration or the development of lesions in the carapace (Bullis, 1994; Nolan and Smith, 2009). These lesions have been associated with a variety of microorganisms including heterotrophic bacteria (e.g., Thompson et al., 2011), *Cyanobacteria* (Leibovitz, 1986), fungi (Tuxbury et al., 2014), and green algae (Braverman et al., 2012). A study focusing on two species of wild, adult Asian horseshoe crabs, *Tachypleus gigas* and *Carcinoscorpius rotundicauda*, isolated bacteria from the gills and mouth, which were identified as members of the genera *Pseudoalteromonas*, *Vibrio*, and *Photobacterium* (Ismail et al., 2015). However, no systematic studies have explored the microbiomes of wild or captive adult horseshoe crabs using cultivation-independent techniques.

The main objective of this study was to document the microbiomes of wild, adult horseshoe crabs at several body locations and track shifts in their microbiomes associated with captivity. We further extended the study by examining the same body locations on several long-term captive animals (>2 years) from the same tank that displayed symptoms of shell disease. From this, we hoped to identify possible symbionts or commensals of a wild horseshoe crab microflora as well as potential pathogens that develop *ex situ*. Our study revealed highly diverse microbial communities in the carapace, cloaca, gills, and oral cavity of wild animals and documented a rapid and steep decline in microbial richness and near-complete restructuring of the microbial community following captivation. The opportunistic pathogens *Aeromonas* (>60%) and *Pseudomonas* (>20%) together comprised >80% of the microbiomes of animals acclimated to the aquarium for over 2 years. In contrast, the cloaca of the tank-acclimated animals was distinct and more diverse, with abundant populations of *Aeromonas*, *Shewanella*, *Vagococcus*, and *Enterococcus*. This study forms a baseline for both wild and captive adult horseshoe crabs and provides a timeline and body atlas to track microbiome shifts associated with captivity. Potential mechanisms for maintenance of wild and diverse microbiota, and their potential importance in health, are discussed herein.

## Materials and Methods

### Sampling and Experimental Design

Three wild animals and native sediments were collected from the wild and dry-shipped overnight by Dynasty Marine (Marathon, FL, United States). Upon receipt, the carapace, book gills, oral cavity, and cloaca were sampled with sterile swabs. The naïve individuals were then uniquely identified by attaching a PIT tag to the underside of their carapace with epoxy and introduced into the public touch-tank exhibit at the Shark Reef Aquarium at Mandalay Bay Resort and Casino in Las Vegas, Nevada. The native sediment was initially collected using a sterile collection cup and subsampled for DNA extraction upon receipt. At 1, 9, and 14 months following introduction to the touch tank, the same animals were sampled at the same four locations using the same protocol as described above. One wild-caught animal’s cloaca sample was not collected at the 1-month time point. Prior to introducing the naïve individuals into the touch tank, the substrate at the bottom of the tank and three tank-acclimated horseshoe crabs (>2 years in captivity) were sampled at the same body locations and using the same protocol to establish a baseline of the microbial community already present. Swabs and the substrate sample were immediately taken to the lab for DNA isolation, described below. Due to a laboratory error during DNA isolation, one tank-acclimated horseshoe crab’s gill sample was not further processed. A table containing relevant information about each sample has been included in Supplementary Table S1.

### Captive Conditions

Captive horseshoe crabs were maintained in the public touch-tank exhibit at the Shark Reef Aquarium at Mandalay Bay Resort and Casino (Las Vegas, NV, United States) during the study. The tank is 2,500 gallons with a water depth of 34 and 4 cm of fine aragonite sand for substrate. The water is held at 23°C, pH 8.1, and a salinity of 33 ppt using Instant Ocean (Blacksburg, VA, United States). The light cycle is 16 h of dim indirect ambient lighting similar to dusk conditions and 8 h of dark. Filtration includes pressure sand-filters, foam fractionator, and a trickle de-gassing bio-filter with twice weekly filter backwashes and 10% water changes. Horseshoe crabs are fed a combination of earthworms, clams, Superba krill, and oysters 6 days per week. Any uneaten food is immediately removed from the tank. Up to nine individual horseshoe crabs occupied the tank during the present study. Other species that share the tank include coral catsharks, *Atelomycterus marmoratus*, and several genera of rays such as *Neotrygon*, *Glaucostegus*, *Urobatis*, and *Trygonorrhina*. Photos demonstrating the extent of lesion development in horseshoe crabs from the touch-tank exhibit have been included in the supplement; these do not depict the exact crabs sampled in this study but are representative of the diseased state of the captive horseshoe crabs (Supplementary Figure S1).

### 16S rRNA Gene Amplicon Sequencing

DNA was extracted from horseshoe crab samples taken using sterile swabs and substrate samples (all stored at -80°C) using the FastDNA SPIN Kit for Soil (MP Biomedicals, Santa Ana, CA, United States) according to manufacturer’s instructions. For both the native and Shark Reef substrate samples, 500 mg of sediment was subsampled from their respective sterile collection cups for the DNA extraction. The V4 region of the 16S rRNA gene was amplified and sequenced using the updated Earth Microbiome Project (EMP) bacterial- and archaeal-specific 515F/806R primer set (Apprill et al., 2015; Parada et al., 2016; Thompson et al., 2017). Amplification, library preparation, and paired-end sequencing were performed at Argonne National Laboratory (Lemont, IL, United States) on an Illumina MiSeq platform (2 × 151 bp) following the standard EMP protocol^1^.

### Sequence Processing, Quality Control, and Data

Paired-end Illumina MiSeq reads were quality filtered, aligned, and assigned to amplicon sequence variants (ASVs) using DADA2 (Callahan et al., 2016) via Qiime2 version 2019.1 (Caporaso et al., 2010; Bolyen et al., 2019). ASVs were classified in Qiime2 using a naïve-Bayesian classifier (Bokulich et al., 2018) trained on the V4 region of the Silva NR99 132 alignment (Pruesse et al., 2007). Any sequences classified as chloroplast or mitochondria as well as those unclassified at the domain level were removed from the analysis.

### Microbial Community Data Analysis

ASVs and Silva-based taxonomy were exported from Qiime2 to be analyzed using R. ASV counts were normalized to account for variable sequencing depth between samples. Alpha diversity indices (Faith’s PD, Observed, Shannon, and InvSimpson) were calculated using R packages phyloseq version 1.28.0 (McMurdie and Holmes, 2013) and picante version 1.8 (Kembel et al., 2010) from ASV counts rarified to an even depth of 2005 SVs. All other analyses used ASV counts scaled to counts-per-million (cpm). The phylogenetic tree used to calculate UniFrac and Faith’s PD diversity metrics was generated using FastTree (Price et al., 2009, 2010) in Qiime2 with the developer-recommended settings. Between-sample dissimilarity was calculated using the Bray-Curtis algorithm implemented in R package vegan version 2.5-6 (Dixon, 2003). Ordination was performed using non-metric multidimensional scaling (NMDS) via the R packages phyloseq and vegan. ASVs were agglomerated at the family-level using the tax_glom function from phyloseq and regressed against each distance matrix using the envfit function of vegan. Taxonomic vectors representing the bacterial and archaeal families that were significantly (*p* < 0.05) correlated with community dissimilarity between samples, as determined by envfit, were displayed on the NMDS plot.

To analyze the similarity in ASV composition over time in captivity, the community matrix was first separated by body location. Then within each body location grouping, the number of ASVs unique to a time point and shared among the time points was calculated and plotted on a Venn diagram using a custom R script (github repository hedlundb/LP16S). To further understand which specific ASVs were changing over time in captivity, differential abundance analysis was conducted using DESeq2 version 1.24.0 (Love et al., 2014). Differentially abundant ASVs of *p* < 0.01 and *p* < 0.05 were aligned using the SINA alignment tool (Pruesse et al., 2012). A phylogenetic tree was constructed from these using IQ-Tree 1.6.7.a (Minh et al., 2013; Nguyen et al., 2015; Kalyaanamoorthy et al., 2017), rooted at its midpoint using phytools 0.6.60 (Revell, 2012), and ladderized using ape 5.3 (Paradis et al., 2004). All figures were rendered using Microsoft PowerPoint, the R package vegan version 2.5-6 (Dixon, 2003), or the R package ggplot2 version 3.2.1 (Wickham, 2011).

## Results

### Rarefaction Curves and Community α-Diversity

Following quality filtering, 1,758,652 total DNA sequences were recovered from 61 samples (59 horseshoe crab samples and two substrate samples). Of these >1.7 million high-quality DNA sequences, 6,844 unique amplicon sequence variants (ASVs) were identified, comprising 64 bacterial and archaeal phyla. Rarefaction curves for all samples plateaued at a reasonable sequence depth (∼5,000 sequences), indicating adequate coverage of the total diversity (Supplementary Figure S2).

**FIGURE 1:**
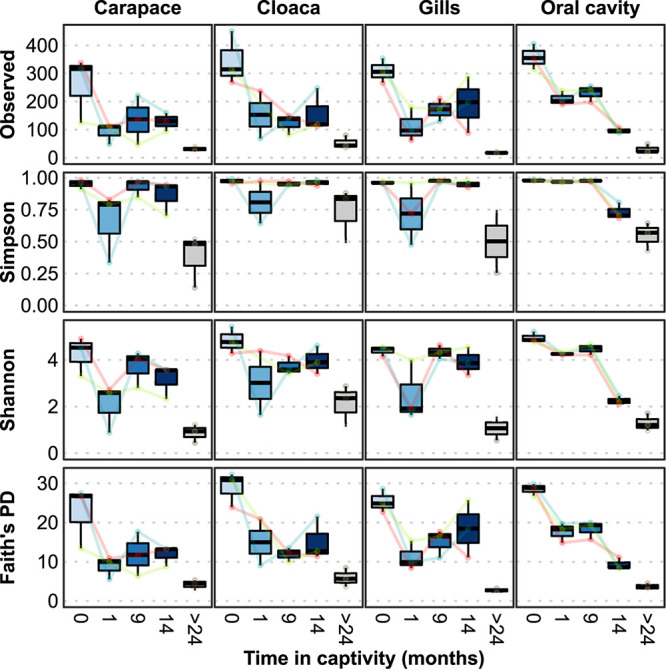
Microbial alpha diversity measures at different locations from wild-caught *Limulus polyphemus* (0 months) introduced to a captive environment and sampled after 1, 9, and 14 months of captivity. Tank-acclimated (>2 years) individuals were sampled prior to introducing the wild-caught individuals into the tank to establish the microbiome already present in the tank. Each colored line represents one of the three wild individuals that was followed over time.

To evaluate the effect of captivity on their microbiomes, several α-diversity indices were calculated at 1, 9, and 14 months in captivity (Figure 1). At all body locations, the wild horseshoe crabs hosted highly diverse microbial communities, with the mean observed ASVs exceeding 300 and Simpson’s evenness above 0.98. After 1 month in captivity, a decline was observed for all diversity indices, indicating a loss of richness (observed ASVs), diversity (Simpson and Shannon indices), and phylogenetic diversity (Faith’s PD). The decline was most clear in the observed ASVs and Faith’s PD index at all body locations and richness did not recover through the end of the study, except for partial recovery in the gills of two animals. A similar pattern of severe decline in Simpson’s Evenness was observed after 1 month for the carapace and gill samples; however, these values returned to near-normal levels within 9 months and remained high through the end of the study. The oral cavity samples displayed a progressive loss of richness over the course of the experimental timeline; evenness remained high but started to decline 14 months after captivity. Throughout the 14 months of captivity, the richness and evenness of the wild horseshoe crab microbiomes never reached the drastically low values of the tank-acclimated population that was in captivity for over 2 years, which had <60 observed ASVs at each body location on average.

### Community Composition of Horseshoe Crabs and Substrate

Both wild and tank-acclimated horseshoe crab microbiomes were dominated by *Bacteria* (>99% of ASVs), whereas *Archaea* were more abundant (∼3–6%) in the Florida and Shark Reef touch tank substrate samples (Supplementary Figure S3). The Florida substrate was the most diverse of all the samples and was comprised of *Proteobacteria*, *Bacteroidetes*, *Chloroflexi*, *Planctomycetes*, *Spirochaetes*, *Cyanobacteria*, *Calditrichaeota*, *Acidobacteria*, and *Actinobacteria* (Supplementary Figure S4). In comparison, the Shark Reef substrate sample was primarily comprised of *Proteobacteria*, *Bacteroidetes*, *Actinobacteria*, *Nitrospirae*, *Thaumarchaeota*, *Acidobacteria*, and *Planctomycetes*. The phylum- and class-level composition of the wild horseshoe crab microbiomes at the four body locations was highly similar, being dominated by *Proteobacteria* (*Gammaproteobacteria* and *Alphaproteobacteria*) and *Bacteroidetes* (*Bacteroidia*), with individual horseshoe crabs possessing varying abundances of *Planctomycetes*, *Patescibacteria*, *Verrucomicrobia*, and *Actinobacteria*. The phylum- and class-level composition of the wild horseshoe crab microbiome was generally retained throughout captivity, with *Gammaproteobacteria, Bacteroidia*, and *Alphaproteobacteria* being the most abundant classes across nearly all body locations and sampling times. However, over the course of 14 weeks in captivity, transient increases in the relative abundance of *Firmicutes*, *Tenericutes*, *Epsilonbacteraeota*, *Fusobacteria*, and an unclassified bacterial phylum were observed, during which these phyla comprised 8.0% to >70% of the microbiome in individual samples. The majority of the tank-acclimated horseshoe crab samples (9/12) were dominated by *Gammaproteobacteria* (>90% of the total community composition). The other three, all cloaca samples, also contained abundant *Firmicutes*, *Bacteroidetes*, and *Epsilonbacteraeota*. Bar plots showing the relative abundance at the domain, phylum, class, order, family, and genus levels for all samples can be found in Supplementary Figures S3–S8.

**FIGURE 2:**
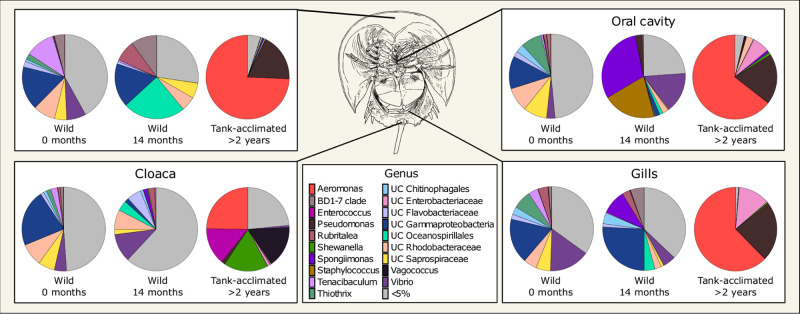
Mean normalized abundance (*n* = 3) of the most abundant genera from different body locations of *Limulus polyphemus*. Wild-caught animals were sampled in the field (0 months) and following 14 months in captivity. For simplicity, samples taken at one and 9 months are not shown. The tank-acclimated individuals had been in the tank for over 2 years but were sampled at the same time the wild-caught 0-month samples were taken to establish the microbiome already present in the tank. Colors represent genera representing >5% of the total microbiome, while gray represents all the uncommon taxa (each < 5%) combined.

Describing phylum-level composition is useful in creating a broad picture of a community and is common practice in the literature, however, it is difficult to extract meaningful information about ecological functions at that taxonomic level. To address this, we explored lower taxonomic levels (Figure 2 and Supplementary Figure S8). The four body locations sampled on the wild horseshoe crabs had highly similar microbial communities, with the most abundant members (at least 50% of the total community) being unclassified members of the *Gammaproteobacteria*, *Chitinophagales*, *Rhodobacteraceae*, *Saprospiraceae*, *Flavobacteriaceae*, and BD1-7 clade, along with the genera *Vibrio*, *Tenacibaculum*, *Thiothrix*, and *Rubritalea* (Figure 2).

Although some dominant genera were retained in the wild-caught population after 14 months in captivity, there were large shifts in community composition, and a divergence of microbial community structure by body location (Figure 2). All body locations were colonized by unclassified *Oceanospirillales* and had a complete loss of *Thiothrix* and decreased abundance of *Tenacibaculum* and unclassified *Rhodobacteraceae* through time. The carapace and cloaca microbial communities had an increased abundance of the genus *Rubritalea*. *Spongiimonas* was observed in the oral cavity, cloaca, and gills, but not the carapace. The composition of the oral cavity samples was drastically different than the initial sample, with an increased abundance of *Vibrio* and an appearance of *Staphylococcus*. Cloaca samples had an increased abundance of *Vibrio* and unclassified *Flavobacteriaceae* and a decreased abundance of unclassified *Gammaproteobacteria*. Gill samples showed an increased abundance of unclassified *Gammaproteobacteria*, *Flavobacteriaceae*, and BD1-7 clade and a decrease in the abundance of *Vibrio*. The microbiome was dynamic over the course of the experimental timeline, with several taxa dominating the community (>10% of the total community) at different times, such as *Shewanella*, *Chromohalobacter*, *Pseudomonas*, *Cocleimonas*, *Spongiimonas*, and *Staphylococcus* (Supplementary Figure S8).

*Aeromonas* and *Pseudomonas* dominated the microbial communities of tank-acclimated carapace, oral cavity, and gill samples, comprising more than 75% of the total community (Figure 2). Additionally, *Cocleimonas* was observed on the carapace and unclassified *Enterobacteriaceae* in the oral cavity and gills (Supplementary Figure S8). The cloaca microbial communities were distinct and more diverse than the other body locations, with *Aeromonas*, *Shewanella*, *Vagococcus*, *Enterococcus*, *Lactococcus*, and *Proteus* present.

### Community Dissimilarity Analysis

**FIGURE 3:**
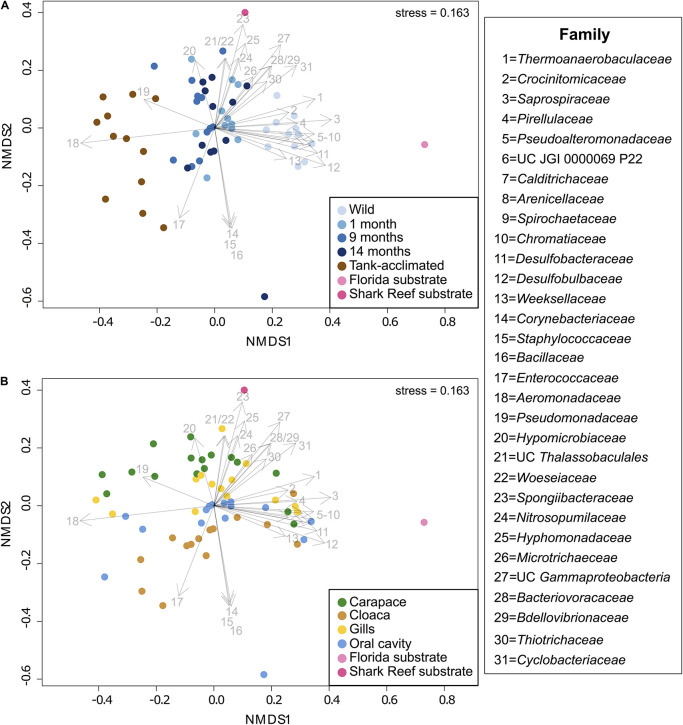
**(A)** NMDS showing the separation of microbiomes from combined sampling locations over time in captivity along NMDS1, including three wild-caught individuals at different sampling times (blue shades) and three tank-acclimated animals (brown; >2 years in captivity). For reference, substrates from the captive habitat and representative sediments from where the wild caught individuals were captured are shown (pink shades). **(B)** NMDS showing the separation of microbiomes from combined sampling times by body location along NMDS2. Taxonomic vectors representing bacterial and archaeal families that were significantly (*p* = 0.05) correlated with community dissimilarity were overlaid on to both plots.

A NMDS analysis of Bray-Curtis dissimilarity for all samples enabled visualization of differences in microbial community composition by time and body location (Figure 3). The distinct microbial communities observed across time and body location were significantly different via PERMANOVA (*p* = 0.001 for both); individual was not a significant factor (*p* = 0.124). Samples separated by time along the x-axis (NMDS1) and body location along the y-axis (NMDS2). There was an increasing dissimilarity between the horseshoe crabs following time in captivity, shown by movement from right to left in the NMDS plot (Figure 3A). Both substrate samples were distinct from all horseshoe crab samples and each other, yet the wild horseshoe crab and captive horseshoe crab microbiomes (1–14 months) were most closely related to the Florida and aquarium substrates, respectively, suggesting environmental filtering. The dysbiotic tank-acclimated animal microbiomes were distinct and distant from both substrate microbial communities. As noted previously, the community composition of wild horseshoe crabs was highly similar across all body locations, and it became increasingly structured by body location during captivity, as evidenced by increased distance between points from right to left in the NMDS (Figure 3B). In striking contrast to the initial samples from the wild-caught animals, the community composition of the tank-acclimated horseshoe crabs was highly dissimilar between individuals, yet still retained some structuring by body location.

To gain a deeper understanding of the taxonomic changes associated with captivity and body location, taxonomic vectors representing the bacterial and archaeal families that were significantly (*p* ≤ 0.05) correlated with community dissimilarity between the samples were fitted onto the NMDS ordination (Figure 3). Unique marker taxa were associated with the different horseshoe crab populations and sediment samples. *Pirellulaceae* (#4), *Pseudoalteromonadaceae* (#5), unclassified JGI 0000069P22 (#6), *Desulfobacteraceae* (#11), *Desulfobulbaceae* (#12), *Weeksellaceae* (#13), *Nitrosopumilaceae* (#24), and *Thiotrichaceae* (#30) were all present in the wild horseshoe crabs but were either partially or completely lost during captivity. Several families were observed at a low abundance in the wild horseshoe crabs but increased significantly during captivity, including *Hyphomicrobiaceae* (#20), *Spongiibacteraceae* (#23), *Hyphomonadaceae* (#25), *Microtrichaeceae* (#26), unclassified *Gammaproteobacteria* (#27), *Bacteriovoracaceae* (#28), and *Bdellovibrionaceae* (#29). After 14 months in captivity, the oral cavity of one wild-caught animal hosted dominant populations of *Corynebacteriaceae* (#14), *Staphylococcaceae* (#15), and *Bacillaceae* (#16). The tank-acclimated animals were dominated by *Enterococcaceae* (#17), *Aeromonadaceae* (#18), and *Pseudomonadaceae* (#19). Several families were associated with the native Florida substrate including *Thermoanaerobaculaceae* (#1), *Calditrichaeceae* (#7), *Spirochaetaceae* (#9), *Chromatiaceae* (#10), *Desulfobacteraceae* (#11), *Desulfobulbaceae* (#12), and *Cyclobacteriaceae* (#31). The touch tank substrate included unclassified *Thalassobaculales* (#21), *Woeseiaceae* (#22), *Spongiibacteraceae* (#23), *Nitrosopumilaceae* (#24), *Hyphomonadaceae* (#25), *Microtrichaeceae* (#26), and *Cyclobacteriaceae* (#31).

### Differential Abundance Analysis of Sequence Variants

**FIGURE 4:**
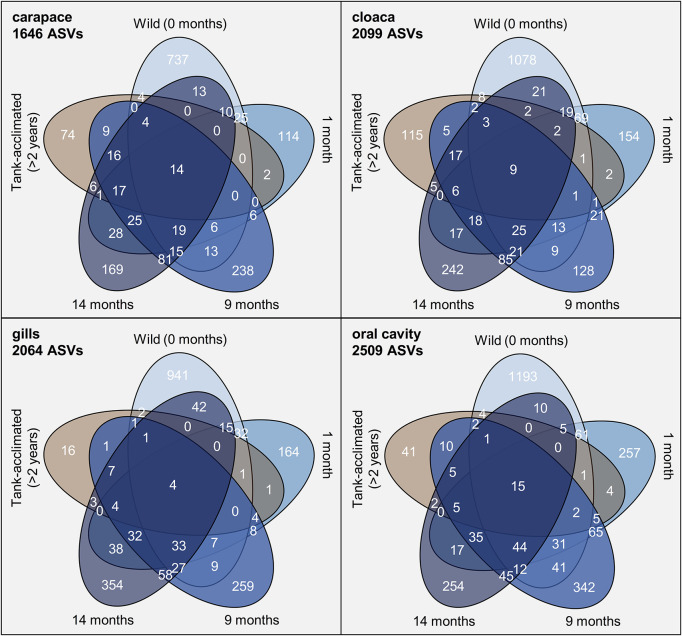
Venn diagrams of the number of amplicon sequence variants (ASVs) identified from different sampling locations on *Limulus polyphemus* over time. In blue, beginning at the top of each diagram is wild-caught prior to captivity (0 months) and moving clock-wise around the diagram are samples from the same individuals at 1, 9, and 14 months of captivity. The final ellipse is from tank-acclimated individuals (>2 years) sampled at the beginning of the study to establish the microbiome already present in the tank.

To more deeply resolve the shifts occurring in the microbial community structure of wild horseshoe crabs as they transitioned to captivity, we conducted several ASV-focused analyses. First, the number of ASVs exclusive to and shared between timepoints was compared (Figure 4). The ASV composition of each time grouping was largely unique (>80%), with very little overlap between time points or between the wild and tank-acclimated populations. Very few ASVs were present throughout the entire study (0.2–0.6%), underscoring the dynamic nature of the microbiome during the transition to captivity. Thus, even though the broader taxonomic structure (i.e., phylum and class) of the wild horseshoe crab microbiome was retained throughout captivity, the microbiome was nearly completely restructured at the ASV level. The loss of diversity evident in the alpha diversity measurements (Figure 1) was also evident here, as shown by the large proportion of unique ASVs in the wild-caught population (44–51% of the total ASV composition) and the decline of unique ASVs over time in captivity. The tank-acclimated horseshoe crab population had the lowest number of unique ASVs (0.8–5.5% of the total ASV composition).

**FIGURE 5:**
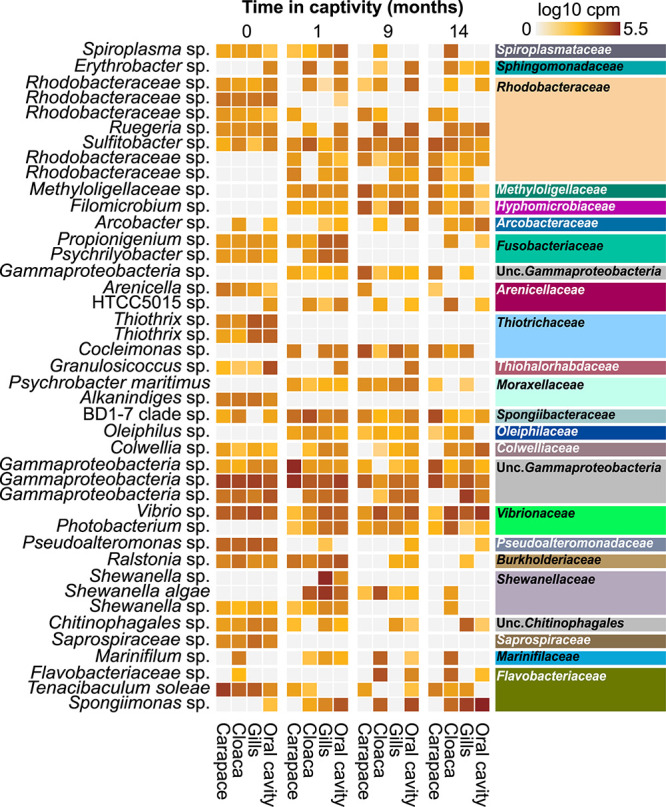
Heatmap showing the log10 counts per million (cpm) of 16S rRNA gene ASVs deemed significantly differentially abundant across time in captivity in the wild horseshoe crabs via DESeq2 analysis (*p* < 0.01). Samples have been grouped initially by time (0, 1, 9, and 14 months), then by body location (carapace, cloaca, gills, and oral cavity). The tip order of the *p* < 0.01 ASV tree was used to order the ASVs phylogenetically. ASVs are labeled according to genus-level taxonomy, indicated on the left. Family-level taxonomy of each ASV is indicated on the right side; colors match family-level bar plot (Supplementary Figure S7).

Differential abundance analysis was employed to identify ASVs that were significantly (*p* < 0.01) differentially abundant in the wild-caught population between sampling locations or through time in captivity (Figure 5). Several ASVs completely disappeared from the wild horseshoe crab microbiome in captivity, including unclassified species of *Thiothrix*, *Psychrilyobacter*, *Granulosicoccus*, *Alkanindiges*, and *Saprospiraceae*. Other ASVs were present in all body locations at the first time point but only partially retained at a lower overall abundance and/or in less body locations after 14 months in captivity, such as *Tenacibaculum soleae* and unclassified species of *Propionigenium*, *Spiroplasma*, *Pseudoalteromonas*, and *Ralstonia*. Additionally, several ASVs associated with *Psychrobacter maritimus* and unclassified species of *Photobacterium*, *Oleiphilus*, *Spongiimonas*, *Cocleimonas*, *Filomicrobium*, *Arcobacter*, and *Arenicella* either increased in abundance or appeared *de novo* during captivity. Various ASVs associated with unclassified members of *Rhodobacteraceae* were abundant in the wild horseshoe crab microbiome but decreased in abundance and/or prevalence or disappeared completely after time in captivity. After 1 month in captivity, new ASVs associated with unclassified members of *Rhodobacteraceae* appeared in the wild horseshoe crab microbiome. Although an ASV associated with an unclassified species of *Shewanella* was initially observed in the wild population, this ASV was partially lost in captivity and replaced by two other *Shewanella* ASVs, another unclassified species and *Shewanella algae.* An additional version of this figure with a more lenient *p*-value cut-off (*p* = 0.05) is available in Supplementary Figure S9.

## Discussion

### Environment Plays a Role in Shaping Microbiome of Horseshoe Crabs

The genera *Pseudoalteromonas*, *Vibrio*, and *Photobacterium* have been previously isolated from book gills and mouth samples collected from adult wild and lab reared Asian horseshoe crab species *T. gigas* and *C. rotundicauda* (Ismail et al., 2015). Although those studies focused on different genera of horseshoe crabs inhabiting geographically distant environments, *Vibrio* and *Pseudoalteromonas* were also detected at appreciable abundance in wild *L. polyphemus*, suggesting they may be part of a natural core horseshoe crab microbiome. *Photobacterium* was also present, however, it was not abundant (<1%) in the microbiomes of wild *L. polyphemus* that we sampled. These three genera are common marine bacteria globally and include known symbionts and commensals of a variety of marine fauna. *Pseudoalteromonas* species are commonly found in the microbiome of different aquatic organisms, including coral species (Shnit-Orland et al., 2012), crayfish (Skelton et al., 2017), and copepods (Moisander et al., 2015). *Vibrio* is commonly found in the microbiomes of aquatic organisms and some species have been identified as pathogens, commensals, and mutualists, yet, an understanding of the relationships between many *Vibrio* species and their hosts requires further research (Thompson et al., 2016). A more comprehensive view of a core adult horseshoe crab microbiome awaits cultivation-independent surveys of other horseshoe crab species, and more individuals from different geographic and physicochemical environments (e.g., estuarine and marine).

Although little research has been done regarding the microbial communities in horseshoe crabs, extensive work has been carried out for other arthropods. A recent *Proteobacteria*-focused meta-analysis of previous arthropod microbiome studies listed ∼500 genera present in the microbiomes of three or more arthropods (Degli Esposti and Romero, 2017). Strikingly, only four of these genera were present at high abundance (>5%) in any of the wild horseshoe crabs, possibly reflecting the predominantly terrestrial nature of many arthropods that have been studied and/or the distant phylogenetic relationship between horseshoe crabs and arthropods included in the meta-analysis. These four genera have been described variably in ticks (a fellow arachnid), shrimps, and/or prawns (fellow aquatic arthropods). For example, *Pseudoalteromonas* was prevalent in the microbiomes of wild horseshoe crabs, ticks, and shrimps. *Thiothrix* was seen in horseshoe crabs and ticks but not in the other groups. *Vibrio* was present in all groups but was most abundant in shrimp and ticks. A high abundance of unclassified members of *Rhodobacteraceae* was observed in both horseshoe crabs and shrimp but not in the other two groups, indicating the prevalence of unclassified members of this lineage in marine arthropods. Strikingly, none of the major genera overlapped between scorpions and horseshoe crabs, despite their phylogenetic relationship within the *Arachnida*. Non-proteobacterial members of the wild-caught horseshoe crab microbiome are similarly not commonly reported in terrestrial arthropods (Otani et al., 2014; Vanthournout and Hendrickx, 2015; Bouchon et al., 2016; Degli Esposti and Romero, 2017).

The results of the meta-analysis support insights from previous studies that the proteobacterial microbiome of terrestrial arthropods is dominated by taxa commonly found in soil communities, implying that members of the microbiome are selectively filtered from an environmental pool (Degli Esposti and Romero, 2017). By extension, horseshoe crabs could be expected to host predominantly marine and estuarine microorganisms rather than soil microorganisms, but only a few taxa were detected in both the wild-caught horseshoe crab and native Florida substrate sample. However, this is not surprising given the environmental heterogeneity of horseshoe crabs, which provides them with a large pool of environmental microbes to interact with in comparison to our one native substrate sample. Generally, this environmental filtering hypothesis was supported by our data, as we observed many microbes commonly associated with marine systems in the wild horseshoe crab microbiomes. Marine systems are typically dominated by *Alphaproteobacteria*, *Gammaproteobacteria*, and *Bacteroidia* (Louca et al., 2016) and those same three classes often predominate in the microbiomes of marine animals (Colston and Jackson, 2016; Thomas et al., 2016; Degli Esposti and Romero, 2017).

Some of the microbes in the wild horseshoe crabs reflect their benthic lifestyle and suggest cycling between oxic and anoxic sediments. In particular, the presence of sulfate-reducing bacteria (SRB) (*Desulfobacteraceae* and *Desulfobulbaceae*) on the wild horseshoe crabs is likely enabled by burrowing or foraging in anoxic sediments where SRB predominate; the digestive system of arachnids is permeable to oxygen and not likely to shield SRB from an oxic environment (Lozano-Fernandez et al., 2016). Similarly, the high abundance and diversity of sulfide-oxidizing bacteria (SOB) in the genus *Thiothrix* in wild animals is consistent with the presence of SRB-derived sulfide. Symbioses between sulfur-oxidizing microorganisms and aquatic invertebrates are widespread in sulfide-rich marine environments and thought to have evolved independently in many organisms (Dubilier et al., 2008; Dattagupta et al., 2009; Distel et al., 2017; Bergin et al., 2018). *Thiothrix* is a common ectosymbiont of marine invertebrates (Temara et al., 1993; Brigmon and De Ridder, 1998; Bauermeister et al., 2012). The genus *Granulosicoccus* was prevalent in the oral cavity samples of the wild horseshoe crabs and is present in the microbiomes of several other marine organisms, such as seagrass (Kurilenko et al., 2010), kelp (Bengtsson et al., 2012), and corals (van Bleijswijk et al., 2015). A genomic analysis of the type strain *Granulosicoccus antarcticus* IMCC3135T (=KCCM 42676^*T*^ = NBRC 102684^*T*^) revealed the presence of several genes associated with sulfur cycling; for example, the genome contained a gene encoding for the enzyme dimethylsulfoniopropionate (DMSP) demethylase and several genes associated with sulfur oxidation (Kang et al., 2018). These bacteria are likely responsible for a complex sulfur biogeochemical cycle in and on wild horseshoe crabs.

### Significant Shifts Associated With Captivity Observed in Microbial Community Diversity and Structure of Horseshoe Crabs

Public aquaria are popular attractions that are commonplace all around the world as stand-alone entities or as additions to museums, malls, and hotels. Taking advantage of their popularity, modern-day accredited aquaria offer an exciting opportunity for the public to view and interact with various aquatic organisms, while also educating attendees about serious environmental issues, such as conservation. Touch-tank exhibits, which are commonly found in aquaria, provide visitors with the chance to directly interact with and touch live aquatic creatures, such as horseshoe crabs, rays, sharks, and others. Given that aquarium staff members are always present at these exhibits, touch tanks provide a unique educational experience beyond that of just attending an aquarium. Although aquaria and touch-tank exhibits are popular attractions worldwide and serve an important role in furthering ecological education, few studies have explored the health of captive organisms (Morris et al., 2012; Persky et al., 2012; Johnson et al., 2017) or described the microbial communities of the aquarium environment and organisms (Patin et al., 2018), how these microbial communities change over time (Van Bonn et al., 2015; Cardona et al., 2018), and how they compare to the natural environment and wild microbiome (Pagán-Jiménez et al., 2019).

An ever-increasing body of literature has demonstrated the importance of diverse microorganisms in ecology, such as biogeochemical cycling (Rousk and Bengtson, 2014), ecosystem health (Hooper et al., 2005), and disease prevention in hosts (Hamdi et al., 2011; Greenspan et al., 2019). In our study, we documented a drastic decrease in the diversity of the wild-caught horseshoe crabs microbiome and significant shifts in microbial community structure shortly after entering captivity. The diversity and composition of the natural microbiome that was lost following entrance into captivity never recovered. The tank-acclimated population, which had spent more than 2 years in captivity, was marked by a highly uneven and low-diversity microbiome. Similar patterns of declines in microbial diversity and microbial community shifts associated with captivity have been observed in sea cucumbers (Pagán-Jiménez et al., 2019) and dugongs (Eigeland et al., 2012). Another study, focused on smooth dogfish, found that there was a high incidence of mortality in adults and pups of an aquarium collection following entrance into captivity, despite various treatments (Persky et al., 2012). Contrastingly, a study assessing the health of cownose rays in a touch-tank exhibit at the John G. Shedd Aquarium to that of a population in an off-exhibit habitat demonstrated that there was no discernible difference in the health of the two populations (Johnson et al., 2017).

Aquarium systems attempt to recreate the natural habitat, but they differ from the natural ecosystem in several ways, including: (1) unnatural physical or chemical conditions (for example, the use of non-native substrate); (2) interactions with organisms that are rarely, if ever, experienced in nature, including microbes seeded from co-habiting species and human contact; (3) an unstable aquarium water microbial community, typically dominated by continuously shifting microbial blooms (Van Bonn et al., 2015; Patin et al., 2018); and (4) increased biomass/population density. We hypothesize that the combined stresses associated with living in a touch-tank exhibit are likely related to the decline in microbial diversity of the horseshoe crabs over time in captivity. Similar loss of microbial diversity associated with stress, such as hypoxia or temperature stress, have been described previously in brook char (Boutin et al., 2013) and Pacific oysters (Lokmer and Wegner, 2015). Another factor that may have contributed to the microbiome shifts observed during captivity in our study is the difference in diet between wild and captive horseshoe crabs. Wild horseshoe crabs encounter a myriad of possible food sources, including dead fish, algae, mollusks, worms, bivalves, and crustaceans. Captive populations, while still receiving a relatively diverse diet, are not exposed to the same level of diversity as their natural counterparts in the ocean. A captive diet limits not only the variety of food sources available, but also the microbial diversity in food inocula, which has been shown to be important in supporting the development of diverse microbial communities (Bolnick et al., 2014; Heiman and Greenway, 2016; Martínez-Mota et al., 2020).

We found that the high-level taxonomy (i.e., phylum and class) of the natural horseshoe crab microbiome was largely retained for over a year, but abundances of individual taxa, such as unclassified members of *Gammaproteobacteria*, *Rhodobacteraceae*, *Flavobacteriaceae*, and *Saprospiraceae*, became increasingly different in all body locations over the course of the experiment. Several unclassified species of *Thiothrix*, an abundant sulfur oxidizer in the wild horseshoe crab microbiome, completely disappeared after only 1 month in captivity and was replaced by unclassified species of a closely related sulfur-oxidizing genus, *Cocleimonas* (Tanaka et al., 2011). Additionally, *Tenacibaculum soleae*, which has been previously identified in diseased aquatic animals and seagrass, was partially lost over time in captivity (Pibñeiro-Vidal et al., 2008; Lujan et al., 2016; Burioli et al., 2018). We also observed the appearance of unclassified species of several novel taxa during time in captivity, including, *Spongiimonas*, *Oleiphilus*, *Oleispira*, and *Oceanospirillales* which we hypothesize to be indicative of the captive environment and seeded from the aquarium water (Van Bonn et al., 2015; Patin et al., 2018). Additionally, we recorded several transient increases in relative abundance of unclassified species of different taxa during captivity, such as *Shewanella*, *Pseudomonas*, *Cocleimonas*, *Corynebacterium*, *Staphylococcus*, and *Spongiimonas*, suggesting a highly unstable and dynamic community following entrance into captivity. Two species previously isolated from marine environments, *Psychrobacter maritimus* and *Shewanella algae*, also found in human clinical samples, appeared and were retained in the horseshoe crab microbiome during captivity (Simidu et al., 1990; Vogel et al., 2000; Romanenko et al., 2004).

Extreme differences between the tank-acclimated and wild-caught horseshoe crab microbiomes were apparent, with the former possessing highly uneven communities dominated by unclassified species of *Aeromonas*, *Pseudomonas*, and *Enterobacteriaceae*, with *Shewanella algae* and unclassified species of *Enterococcus* also abundant in the cloaca. The appearance of several unclassified species of human-associated taxa, such as *Enterococcus*, *Corynebacterium*, *Staphylococcus*, and *Prevotella*, in the captive animal populations may be related to the dynamic nature of the touch-tank exhibit, where various visitors are in direct contact with the animals. This might facilitate the transfer of human-associated taxa to the captive horseshoe crabs. This is in direct opposition to a study on cownose rays in touch tanks, which concluded that the transfer of human-associated taxa to the rays was negligible (Kearns et al., 2017). Cultural analyses of hemolymph samples from captive *Limulus* resulted in the isolation of several bacterial species associated with pathological conditions, including *Shewanella putrefaciens* (formerly *Pseudomonas putrefaciens*) and *Aeromonas hydrophila* (Khashe and Janda, 1998; LaDouceur et al., 2019). We hypothesize that the unclassified species of *Pseudomonas* and *Aeromonas* that dominate our tank-acclimated population are involved somehow in the development of the diseased state in our study. However, this hypothesis was not properly addressed by this study, but could be addressed in the future, for example, by applying principles of Koch’s Postulates.

### Symbiotic Potential of Taxa Identified in the Wild Horseshoe Crab Microbiome

Termites and cockroaches are well-studied arthropod model organisms in symbiosis research and several studies have demonstrated the importance that symbiotic interactions played in their evolution, particularly their expansion into previously unoccupied niches, such as plant polymer degradation (Berlanga and Guerrero, 2016). In addition, marine symbioses are also quite well researched, with various studies detailing the biotechnological potential of and interactions between host-symbiont in deep-sea hydrothermal vents (Zimmermann et al., 2014; Ho et al., 2017), coral reefs (Venn et al., 2008), sponges (Venn et al., 2008), and polychaetes (Goffredi et al., 2005). We speculate that sulfur-cycling microorganisms, such as *Thiothrix* and *Granulosicoccus*, may play an important role in mediating horseshoe crab health and microbiome composition, possibly by competing with pathogens for attachment space and/or nutrients. As discussed previously, these microorganisms would benefit by living at or near redox interfaces and/or by transitioning frequently between oxic and anoxic environments that may source terminal electron acceptors and donors, respectively, and a variety of other nutrients. Additionally, several species of *Pseudoalteromonas* are widely discussed in the literature as symbionts, given their diverse metabolic potential and their ability to produce a variety of antibacterial, antifungal, algicidal, and antifouling compounds (Offret et al., 2016; Atencio et al., 2018).

One pathology associated with captive horseshoe crabs is the appearance of pitting or lesions on the carapace, which is composed primarily of chitin (Nolan and Smith, 2009). Once the exoskeleton of the horseshoe crab has been breached (or degraded), it would then be susceptible to secondary infections and given that adults do not molt, the damage accumulated is irreparable. Previous studies have associated infections in captive lab horseshoe crab populations with eukaryotic parasites, algae, fungi, and bacteria (commonly *Cyanobacteria* and Gram-negative bacteria) (Leibovitz and Lewbart, 2003; Smith, 2007; Braverman et al., 2012; LaDouceur et al., 2019). We hypothesize that the appearance of these lesions could be due to shifts in the horseshoe crab microbiome, resulting in an overall increase in the abundance of chitinolytic bacteria or the appearance of opportunistic pathogens capable of degrading chitin. More specifically, we posit that certain taxa in the wild animals with potential chitinolytic activity, such as *Vibrio* and *Pseudoalteromonas* (Vogan et al., 2008; Machado et al., 2015), may represent commensalistic or mutualistic members of the microbiome that help remove loose material, while out-competing potentially pathogenic chitinolytic bacteria. These organisms are also likely kept in check by other microorganisms in nature. In contrast, we observed highly abundant and potentially pathogenic ASVs of unclassified *Aeromonas* and *Pseudomonas* species in captivity, which could contribute to the development of lesions and disease over time (Frederiksen et al., 2013; LaDouceur et al., 2019; Salighehzadeh et al., 2019). Fungi have also been identified as possible chitinase containing pathogens that could be involved in this process (Tuxbury et al., 2014; LaDouceur et al., 2019). In our study, we excluded eukaryotic sequences from analysis and therefore were unable to document the fungal communities of our samples.

## Conclusion

This study details the first cultivation-independent survey of the adult horseshoe crab microbiome. The microbial community of wild horseshoe crabs was diverse and highly similar across body locations but was nearly completely restructured through time in captivity. Changes in the microbiomes of horseshoe crabs over time in captivity includes significant loss of diversity, increasing uniqueness by body location, dynamic shifts in the most abundant taxa, and eventual development of highly uneven dysbiotic communities dominated by a few opportunistic pathogens, primarily *Aeromonas* and *Pseudomonas*.

This study provides an initial framework for understanding the horseshoe crab microbiome and its response to captivity. We suggest some directions for future study below. Sampling could be increased and expanded. The wild horseshoe crabs that were sampled represent a very small portion of the geographic distribution and physicochemical conditions of *L. polyphemus* in the wild. To more deeply understand the diversity of the natural microbiome of these animals, additional sampling throughout their range and at different points in their life cycle is warranted. A longer experimental timeline (>2 years in captivity) and more frequent sampling could provide a more detailed view of how the microbiota change in captivity. While we found evidence of bacteria that are known to contain chitinase, it is still unknown if the carapace lesions are caused by bacteria or if the lesions develop first, then become opportunistically infected after. A directed investigation that includes progressively analyzing the lesions as they develop or intentionally infecting individuals with different strains of bacteria could shed light on the mechanism of disease. Several bacteria found on captive horseshoe crabs were typical of human hosts and others were known human pathogens—understanding more about the transmission of these bacteria in both directions is important for the health and safety of both the animals and the visitors of the exhibit. Other avenues to explore include understanding the role substrate type and depth plays in microbial associations and how the density of individuals including co-habiting species affect the rate at which the microbial community changes.

## Data Availability Statement

The datasets generated for this study can be found in the NCBI Sequence Read Archive (SRA) under the bioproject accession number PRJNA605038. Scripts for all analyses performed as well as qiime2-compatible archive files are available in the GitHub repository hedlundb/LP16S.

## Author Contributions

SN, JJ, and LB conceived the study and experimental design. SN, LB, FL, GM, AF, and CS collected and processed samples. SN, BH, AF, and CS worked together to design the figures. AF and CS were responsible for generating figures for the manuscript. AF and SN co-wrote the first draft of this manuscript, which was initially edited by BH. All authors contributed to the article and approved the submitted version.

## Conflict of Interest

The authors declare that the research was conducted in the absence of any commercial or financial relationships that could be construed as a potential conflict of interest.

## Footnotes

1 https://earthmicrobiome.org/protocols-and-standards/16s/

## Supplementary Material

FIGURE S1 Representative photos depicting the extent of shell disease in horseshoe crabs from the touch-tank exhibit at the Shark Reef Aquarium. Observations for each individual are as follows: **(A)** Male with bilateral pitting behind lateral compound eyes and dark pit/spot along the median ridge of dorsal cephalothorax. Dark spot on left dorsal opisthosoma near telson base and random pitting and dark pigmentation spread throughout; **(B)** Female with identifiable dark patches on dorsal opisthosoma. Broken 3rd walking leg on left side; **(C)** Male with dark discoloration spread over dorsal cephalothorax with two bilateral lines between lateral compound eyes. Dark line halfway down telson. Two bilateral marks on distal portion of ventral cephalothorax. Missing tip of 4th walking leg; **(D)** Female with large dark spots on dorsal opisthosoma.

FIGURE S2 Rarefaction curve comparing the number of total observed species (Species) to the number of reads in each sample (Sample Size). Samples are colored corresponding to the time series: substrates (pink), wild (shades of blue), and tank-acclimated (brown).

FIGURE S3 Bar plot representing the domain-level relative abundance of taxa in all samples. The two substrate samples have been grouped and all horseshoe crabs have been grouped by individual sampled. Taxonomic classification was based on SILVA (version 132).

FIGURE S4 Bar plot representing the phylum-level relative abundance of taxa in all samples. The two substrate samples have been grouped and all horseshoe crabs have been grouped by individual sampled. Taxa whose abundance was <5% were grouped together and listed as such. All unclassified taxa at this rank and the <5% group are colored gray. Taxonomic classification was based on SILVA (version 132).

FIGURE S5 Bar plot representing the class-level relative abundance of taxa in all samples. The two substrate samples have been grouped and all horseshoe crabs have been grouped by individual sampled. Taxa whose abundance was <5% were grouped together and listed as such. All unclassified taxa at this rank and the <5% group are colored gray. Taxonomic classification was based on SILVA (version 132).

FIGURE S6 Bar plot representing the order-level relative abundance of taxa in all samples. The two substrate samples have been grouped and all horseshoe crabs have been grouped by individual sampled. Taxa whose abundance was <5% were grouped together and listed as such. All unclassified taxa at this rank and the <5% group are colored gray. Taxonomic classification was based on SILVA (version 132).

FIGURE S7 Bar plot representing the family-level relative abundance of taxa in all samples. The two substrate samples have been grouped and all horseshoe crabs have been grouped by individual sampled. Taxa whose abundance was <5% were grouped together and listed as such. All unclassified taxa at this rank and the <5% group are colored gray. Taxonomic classification was based on SILVA (version 132).

FIGURE S8 Bar plot representing the genus-level relative abundance of taxa in all samples. The two substrate samples have been grouped and all horseshoe crabs have been grouped by individual sampled. Taxa whose abundance was <5% were grouped together and listed as such. All unclassified taxa at this rank and the <5% group are colored gray. Taxonomic classification was based on SILVA (version 132).

FIGURE S9 Heatmap showing the log10 counts per million (cpm) of 16S rRNA gene ASVs deemed significantly differentially abundant across time in captivity in the wild horseshoe crabs via DESeq2 analysis (*p* < 0.05). Samples have been grouped initially by time (0, 1, 9, and 14 months), then by body location (carapace, cloaca, gills, and oral cavity). The tip order of the *p* < 0.05 ASV tree was used to order the ASVs phylogenetically. ASVs are labeled according to Genus-level taxonomy, indicated on the left. Family-level taxonomy of each ASV is indicated on the right side; colors match family-level bar plot (Supplementary Figure S7).
